# Counting Small Particles in Electron Microscopy Images—Proposal for Rules and Their Application in Practice

**DOI:** 10.3390/nano12132238

**Published:** 2022-06-29

**Authors:** Harald Bresch, Vasile-Dan Hodoroaba, Alexandra Schmidt, Kirsten Rasmussen, Hubert Rauscher

**Affiliations:** 1 Federal Institute for Materials Research and Testing (BAM), D-12200 Berlin, Germany; schmidtalexandra63@gmail.com; 2 Joint Research Centre (JRC), European Commission, 21027 Ispra, Italy; kirsten.rasmussen@ec.europa.eu (K.R.); hubert.rauscher@ec.europa.eu (H.R.)

**Keywords:** nanomaterial, particle size distribution, electron microscopy, measurement procedures, regulation, nanoparticle, counting rules

## Abstract

Electron microscopy (EM) is the gold standard for the characterisation of the morphology (size and shape) of nanoparticles. Visual observation of objects under examination is always a necessary first step in the characterisation process. Several questions arise when undertaking to identify and count particles to measure their size and shape distribution. In addition to challenges with the dispersion and identification of the particles, more than one protocol for counting particles is in use. This paper focuses on precise rules for the counting of particles in EM micrographs, as this influences the measurement accuracy of the number of particles, thus implicitly affecting the size values of the counted particles. We review and compare four different, commonly used methods for counting, which we then apply in case studies. The impact of the selected counting rule on the obtained final particle size distribution is highlighted. One main aim of this analysis is to support the application of a specific, well-defined counting approach in accordance with regulatory requirements to contribute to achieving more reliable and reproducible results. It is also useful for the new harmonised measurement procedures for determining the particle size and particle size distribution of nanomaterials.

## 1. Introduction

Knowing the size distribution of particulate materials is important for several reasons, especially if such materials are expected to contain very small particles, including particles with sizes at the nanoscale, i.e., of up to around 100 nm (so-called nanoparticles) [1,2,3,4]. A measurement range considerably larger than 100 nm is important for understanding the particle size distribution of the entire particulate material. The considerations in this paper thus apply to the measurement of small particles in general. The physicochemical properties, and as a consequence, also the (bio)chemical reactivity and the (eco)toxicological behaviour, depend on the combination of the chemical nature of the materials, morphological and structural features and their small external dimensions, and may differ considerably between small and larger particles. The particle size distribution, which may be based on mass, volume or particle number, is therefore considered essential knowledge for the safety assessment of particulate material, and there is a general consensus that it should be part of a complete physicochemical characterisation of such materials [5].

In 2009, the OECD Working Party on Manufactured Nanomaterials (WPMN) published the list of end-points to be investigated [6] as possibly relevant for nanomaterials in the WPMN’s Testing and Assessment Programme. One suggested end-point for the physicochemical properties was the particle size and particle size distribution, which was confirmed to be important for identifying nanomaterials [5], also noting that a new test guideline is needed for obtaining the particle size and particle size distribution of nanomaterials.

Information on the particle size distribution of a material is obtained through measurements. The outcomes of these measurements fundamentally depend on what is actually identified and counted as particles. The identification of particles depends on the capability of a specific measurement method to do this [7,8] and, furthermore, the evaluation of a measurement. Hence, the resulting particle size distribution crucially depends on the application of specific counting rules and associated uncertainties. These counting rules are also relevant in a regulatory context, for example, for the new OECD Test Guideline (TG) 125 on Particle Size and Particle Size Distribution of Nanomaterials [9,10]. TG 125 outlines the main methodology to determine particle size distribution and suggests several measurement techniques, including EM, but it does not contain details on how to perform the counting of particles in EM images. This paper is important information complementary to TG 125.

Hence, this paper addresses a sensitive point in the measurement of the particle size distribution, namely the measurement accuracy of the number of particles (usually indicated on the ordinate [Y-axis] of the size distribution), which implicitly affects the size values of the counted particles (usually indicated on the abscissa [X-axis]), i.e., the key parameters of the particle size distribution.

In a regulatory context, the particle size distribution is often required information. Information requirements for registration of chemicals in particulate form according to the European Union (EU) Regulation on Registration, Evaluation, Authorisation and Restriction (REACH) include the particle size distribution, and there are specific information requirements on the particle size distribution for nanomaterials [11]. Likewise, if ingredients in nanoform are intended to be used in cosmetic products, information on the particle size distribution must be submitted to the European Commission (EC) in a notification process [12]. For risk assessment of materials containing small particles, including nanomaterials, intended to be used as food ingredients, the EFSA Guidance on Risk Assessment of Nanomaterials [13] also requires reporting of the particle size distribution.

The United States Food and Drug Administration (US-FDA) has published “Guidance for Industry Considering Whether an FDA-Regulated Product Involves the Application of Nanotechnology” [14]. According to this Guidance, it has to be considered “whether a material or end product is engineered to have at least one external dimension, or an internal or surface structure, in the nanoscale range (approximately 1 nm to 100 nm) and whether a material or end product is engineered to exhibit properties or phenomena, including physical or chemical properties or biological effects, that are attributable to its dimension(s), even if these dimensions fall outside the nanoscale range, up to one micrometer (1000 nm)”. The United States Environmental Protection Agency (US-EPA) has issued a rule for TSCA (Toxic Substances Control Act) Reporting and Recordkeeping Requirements addressing “Chemical Substances When Manufactured or Processed as Nanoscale Materials”. That rule generally applies to chemical substances that are manufactured or processed in a form in which any particles, including aggregates and agglomerates, are in the size range of 1–100 nanometres (nm) in at least one dimension; and that are manufactured or processed to exhibit one or more unique and novel properties [15].

In EU legislation, certain descriptors related to the particle size distribution are used to identify a material as a nanomaterial. The definition of nanomaterial recommended by the European Commission [16] uses the median value of the particle size distribution by number as a criterion to identify nanomaterials: if that median value is smaller than 100 nm, the material is considered a nanomaterial. REACH specifies that a “nanoform” is a substance “…containing particles, in an unbound state or as an aggregate or as an agglomerate and where, for 50% or more of the particles in the number size distribution, one or more external dimensions is in the size range 1 nm–100 nm…” In other words, REACH uses the median value of the particle size distribution as regulatory binding decision criterion whether a substance is a nanoform or not.

If a material is considered a nanomaterial for regulatory purposes, this triggers further regulatory requirements. In the EU, relevant regulatory sectors include but are not limited to chemicals in general (REACH), cosmetics, food, biocides and medical devices [12,17,18,19]. Other jurisdictions around the world have provisions covering the application of nanotechnology and nanoscale materials [20,21].

As described above, knowledge of the particle size distribution is important in a regulatory context. It is furthermore a key property that determines many aspects of material characteristics, and controlling it can be critical to achieving a certain property in a product. The colour of pigments and the catalytic activity of certain particles are two examples where the desired material property depends on the size distribution of its particles.

Among others, the EU 7th framework programme project NanoDefine studied experimentally the available measurement techniques that were candidates for achieving a reliable analysis of the number-based size distribution of particulate material. The outcomes of this study are presented in the NanoDefiner Methods Manual [22], which reviews the capabilities of each method. Part two [8] of this manual is especially relevant as it reviews each technique, including the strengths and weaknesses of each method when measuring nanoparticles. Of the more than 15 measurement techniques studied, EM turned out, overall, to be the best method to characterise nanomaterials.

EM provides a 2-dimensional projection of 3-dimensional particles and is a “counting” method [8], which means that, in contrast to an “ensemble” method, EM measures the size particle-by-particle from an image (micrograph). Hence, the measurement outcome is naturally a number-based size distribution of a well-defined size descriptor, from which other descriptors such as D50 (or other percentiles), mean size or polydispersity can easily be derived. EM is generally considered the gold standard for the characterisation of the size and shape of small particles, including nanoparticles. The limitation of EM is that if beam damage to the material is expected, it should not be used [23], and neither should the technique be used if no clear separation of particles within agglomerates/aggregates is visible. For the accuracy of the results, it is important to note that apart from the applied counting rule, quality criteria such as error budgets, uncertainty and reproducibility always have to be taken into account. However, these issues are not addressed in this publication as we address only the identification of particles and hence corresponding mathematical approaches are not relevant. When performing a full particle size distribution determination, relevant mathematical approaches are described in detail elsewhere, for example, in the ISO Guide 35 on uncertainty, in ISO 21363 and in the new OECD TG 125 Particle size and particle size distribution of nanomaterials [9,24,25].

In order to determine the particle size distribution of a specific sample in practice, several steps are required, each with its own challenges. These steps are: (i) preparation of the sample to be deposited on a proper substrate, (ii) artefact-free image acquisition (e.g., avoiding charging effects), (iii) identification of particles to be counted in the recorded image, (iv) measurement of the particle size, and (v) evaluation of the particle size (and shape) distribution with its key characteristics (type of distribution, mean, median, width). The focus of this paper is on step (iii), for which we propose four ways of counting particles, naming these ways ‘counting rules’. For ease of reference to the counting rules, we propose a well-defined labelling system for them, using the term “counting rule” (CR) and numbering them from one to four (x) and the abbreviation “CRx”.

Hence, in this paper, we discuss the measurement accuracy of the number of particles as it implicitly affects the size values of the counted particles. We give an overview of four different approaches to counting particles, and especially which particles to count and how they are counted when analysing images obtained via electron microscopy. These counting rules are, among others, relevant when analysing the outcomes of testing performed under the new OECD TG 125 on Particle size and Particle Size Distribution of Nanomaterials, as the TG does not contain counting rules. When deciding on which counting rule to apply to results obtained by OECD TG 125, regulatory requirements should also be taken into account. Furthermore, some of the CRs are used in ISO (International Organization for Standardization) documents, and the paper illustrates some of the differences that can arise when applying different counting rules. The particles which are counted in the illustrations and figures are labelled with numbers. Further details can be found in the Supplementary Information.

## 2. Terms, Definitions and Agglomeration State

### 2.1. Primary Particles, Individual Particles, Agglomerates and Aggregates

**Figure 1:**
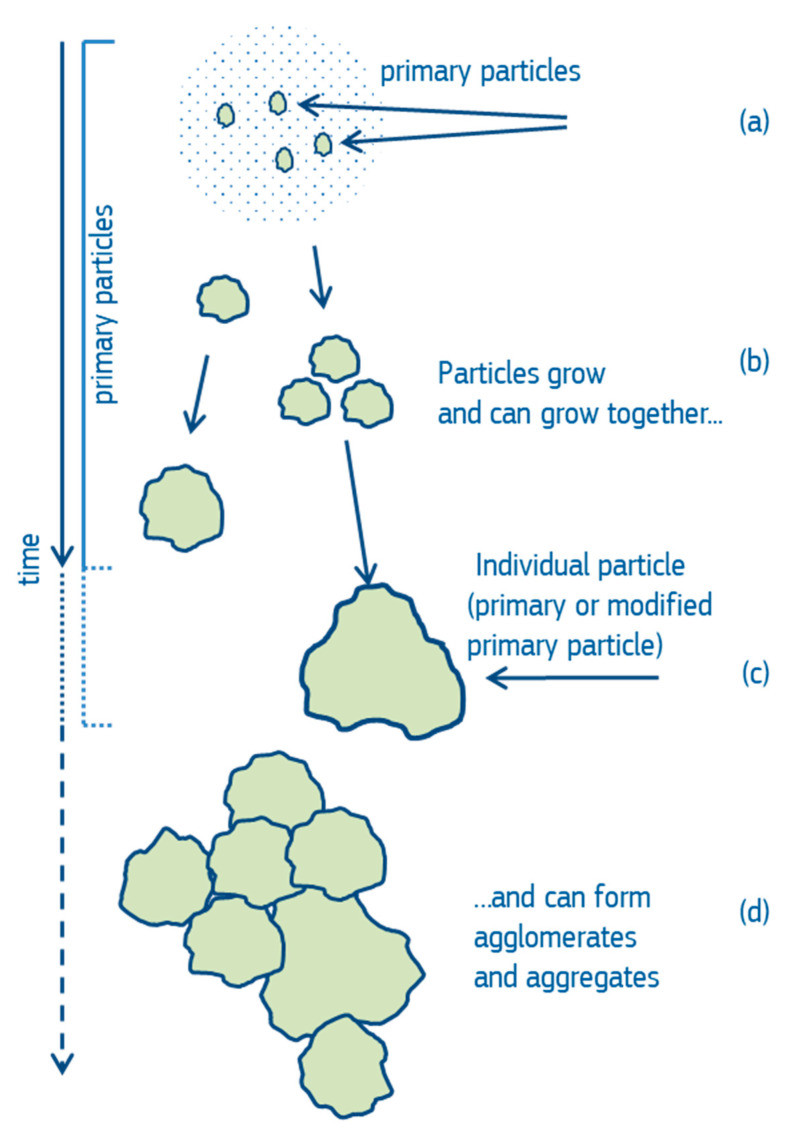
Illustration of particle generation via the bottom-up process.

In order to understand which of the different particles are taken into account by the different counting rules and how they are accounted for, it is useful to review how particles are generated. Particles can be generated by top-down manufacturing processes, e.g., milling of larger starting entities, or by bottom-up processes, e.g., growing from gases, solutions or plasmas. If particles are generated by growth, a seed particle, which can be created through a random nucleation process—or it can also be an impurity—grows in size by the addition of other atoms, molecules or other particles through random impact events (Figure 1a). This results in polycrystalline, monocrystalline or amorphous particles, or mixtures of these, of various sizes and shapes. An ensemble of individual particles emerges, which, in accordance with ISO 80004-2 [26], are called “primary particles” (Figure 1a–c). During their lifetime, primary particles may undergo many changes such as partial dissolution, contamination, degradation or change of the chemical composition and thereafter may be called “modified primary particles” or simply “individual particles” (which also include unmodified primary particles) (Figure 1c). Individual particles may form strong chemical bonds with other particles, resulting in “aggregates”. When the bonds between the particles are weaker, “agglomerates” are formed (Figure 1d). If primary particles agglomerate and subsequently de-agglomerate, they are not primary particles anymore, but they are still “individual particles”. There is no generally agreed quantitative rule on how to differentiate agglomerates from aggregates. The rule of thumb is that the outer surface of aggregates is considerably lower than the sum of surfaces of the former individual particles, whereas the surface area of an agglomerate is similar to the sum of the surface areas of the particles the agglomerate consists of [26].

In the context of describing particulate matter, the term “constituent particle” is often used. Authoritative dictionaries define “constituent” as “That jointly constitute, compose, or make up. Of a single element: That goes to compose or make up; component” [27] and: “serving to form, compose, or make up a unit or whole” [28]. It is therefore clear that in order to understand the exact meaning of “constituent” in a certain context, the point of reference is crucial, i.e., the “unit” or “whole” to which it refers. This is the point where ISO and the EC differ in their interpretation and use of the term “constituent particle”. In the case of the ISO definition, the point of reference is the larger particles or “units”, such as agglomerates and aggregates, whereas, for the EC definition, the point of reference is the initial material or “whole” material. According to ISO 80004-2, a constituent particle is an “identifiable, integral component of a larger particle” [26]. The European Commission, in contrast, formulates the definition of the term nanomaterial: ” ‘nanomaterial’ … should be based solely on the size of the constituent particles of a material…” and continues further explaining what these constituent particles are: “ ’Nanomaterial’ means a … material containing particles, in an unbound state or as an aggregate or as an agglomerate…” [16]. This means that according to the ISO description [26], constituent particles are always part of a larger ensemble and can be aggregated themselves, which could form even larger agglomerates. Following the EC description [16,29], the term “constituent particle” refers to the entire material, so individual particles, as well as the constituents of agglomerates and aggregates, are considered constituent particles of a material. Table 1 summarises the description of ‘constituent particle’ within ISO and in the EC nanomaterial definition.

The counting rules proposed in this work are intended to be applicable in any context where it is important to know the size distribution of particulate matter containing small particles. The actual selection of the relevant counting rule to apply depends on the context and objective of the specific study. It may also be useful to compare the results obtained when applying different counting rules. In order not to introduce any terminological ambiguity here, we will therefore not use the term “constituent particle” in this article. Instead, we use the term “integral component” for the building blocks of agglomerates and aggregates and “individual particle” for particles, which are not part of agglomerates or aggregates and are not agglomerates/aggregates themselves.

### 2.2. Sample Preparation

Nanoparticle samples can be available in different forms for characterisation, e.g., as a powder or as a liquid suspension. For characterisation, the sample needs to be prepared according to the measurement purpose. Often, the aim is to determine the particle size distribution to decide whether the material is a nanomaterial or not, and often, the sample needs to be conditioned to break agglomerates and mainly retain aggregates and individual particles within the sample. This conditioning might be performed by different methods, e.g., by ultrasonication or vortexing, depending on the material properties. Additional to the conditioning, the sample might be transferred into another phase, e.g., from powder to a suspension, from suspension to an aerosol, or from powder to a resin matrix. It should be reiterated that the sample preparation could give rise to artefacts, if not carried out carefully. This paper focusses on the identification of particles to be counted in the recorded image, i.e., the sample preparation is not the subject of this publication. All preparation steps might influence the resulting size distribution and need to be performed and recorded with great care. The following considerations start with the already prepared specimen and will present different cases on how to count the particles. For EM, it is appropriate and usual to distinguish between the “sample” itself (suspended in a liquid, in a solid matrix or as an aerosol) and the sample prepared on a TEM-grid or other substrates for measurements by EM, for which the common term is “specimen” [24]. After the preparation of the specimen there are often particles simply lying beside each other, which are not bound or are only weakly bound to each other. These specific kinds of agglomerates are not part of the original sample and, in this paper, are referred to as “touching particles”.

### 2.3. Agglomeration Cases on the Substrate

As outlined above, standardisation bodies (such as ISO) and legislation have different approaches to identifying and counting individual particles, agglomerates and aggregates.

Nanomaterials prepared for EM measurements will have different agglomeration states. In this paper, we propose five main cases regarding the dispersion state, which have an increasing uncertainty in the resulting size distribution.

(a) An ideally dispersed sample/specimen: A specimen containing only individual particles, separated from each other, which can be counted easily “one-by-one”. The uncertainty for the size distribution pertains to the instrumental resolution and the (subjective) view of the operator.
(b) A predominantly well-dispersed sample/specimen with touching particles: the sample preparation process has led to agglomeration of individual particles on the substrate. If it is known that the sample preparation has resulted in solely individual particles and that agglomeration results only from the preparation of the specimen, the operator can decide which particles are touching and/or overlapping and not completely visible. The uncertainty of the resulting size distribution is greater than in case (a), but in most cases, the counting procedures (a) or (b) will not influence the resulting size distribution.
(c) A predominantly well-dispersed sample/specimen with a few agglomerates: The sample itself contains agglomerates, which could not be separated during the sample preparation process. In this case, it is not possible to distinguish between agglomerates from the sample and touching particles occurring during/after deposition on the substrate. The uncertainty further increases in comparison with case (b). Depending on the choice of rule to identify and count particles, agglomerates and aggregates (by standardisation bodies or from legislation), the particles might be identified and counted differently, possibly leading to significant differences in the resulting size distribution.
(d) A dispersed sample/specimen with agglomerates and aggregates: The specimen contains aggregates and agglomerates predominantly. The distinction between aggregates and agglomerates with electron microscopy is, in most cases, based on assumptions. If no gaps can be identified between the integral components of the agglomerates/aggregates, the particles will probably be identified as aggregates; otherwise, they will be identified as agglomerates. Depending on whether the ISO or regulatory definitions are applied, agglomerates and aggregates might be counted differently, possibly resulting in significantly different size distributions.
(e) A dispersed sample/specimen with agglomerates, aggregates and agglomerates of aggregates: The specimen contains aggregates and agglomerates and agglomerates of aggregates. Depending on whether ISO or regulatory definitions are applied, particle identification and counting might be very different. In practice, the sample preparation needs to be optimised as far as possible, or the counting will be strongly subjective and depend on a combination of sample preparation, used instrument and the operator. This case might result in a specimen/sample that cannot be reliably analysed by EM due to the highly subjective interpretation of the image.

It should be noted that the process of drying a (nano)particle-containing droplet on a substrate for subsequent image analysis with EM involves likely drying artefacts such as agglomeration at the boundary of the droplet. The so-called coffee-ring effect is well-known [30] and hinders a realistic counting of the particles in their original agglomeration state as they were in the initial liquid suspension [31]. Correction procedures are available to keep the agglomeration state of the particles unaltered during drying on a substrate, e.g., pre-treatment of the substrate surface, increased humidity, etc. Complete drying of the solvent and avoiding additional agglomeration during deposition of the particles on a substrate are crucial for the accurate automated analysis of the particle size distribution. Further, the exposure to the high vacuum in the microscope chamber and to the electron beam might also influence the size, shape and agglomeration state of the (nano)particles, especially those with low atomic numbers. Gentle analysis conditions shall be applied. The results obtained from a measurement are strictly valid only for the analysed specimen(s). One should therefore be aware that before drawing conclusions on a larger batch of material, it is necessary to verify that the measured specimen(s) are representative for the batch and that the preparation is reproducible.

## 3. Counting Rules

As noted above, the particle size distribution obtained from the analysis of a particulate material depends directly on what is counted as particles, i.e., on the specific counting rule (CR) applied. In the following, we discuss several possibilities for counting particles, which differ from each other in the way that they take into account individual particles, aggregates and agglomerates. The actual selection of one of these counting rules depends on the context and purpose of characterising a material, which could be, e.g., research, quality control or regulatory purposes. We only use the overarching measurand “diameter”, which might be a substitute for equivalent circular diameter, minimum Feret diameter or maximum Feret diameter, depending on purpose and requirements. A graphical overview of the four counting rules can be found in the Supplementary Information.

### 3.1. Counting Only Individual Particles—CR1

**Figure 2:**
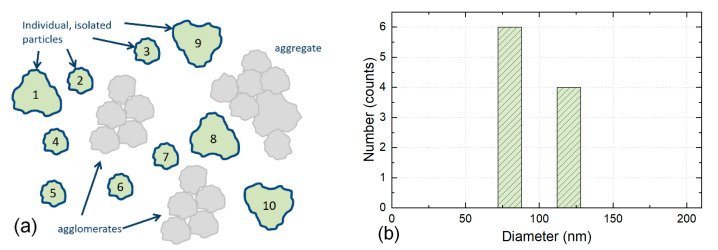
Illustration of Counting Rule 1. (**a**) The particles 1 to 10 are individual particles, without an internal structure that could identify them as agglomerates or aggregates. Only those 10 particles are counted, agglomerates and aggregates (grey) are excluded. (**b**) For the sake of illustration, we assume here only two different types of individual particles, with a size of 80 nm (particles numbered 2 to 7) and 120 nm (particles number 1, 8, 9 and 10). The height of the two columns in the histogram reflects the number of particles of the two sizes 80 nm (6 particles) and 120 nm (4 particles). The width of the columns has no quantitative relevance and is only illustrative.

Agglomerates and aggregates identified in the electron micrographs are excluded from counting, see Figure 2.

The obtained size distribution is based on individual particles only. In order to establish valid statistics, the minimum counting number criteria [24] need to be fulfilled for the identified and counted individual particles. An extrapolation to the entire material works only under the assumption that the aggregates and agglomerates consist of integral components, which have the same size distribution as the individual particles counted.

### 3.2. Counting Individual Particles, Agglomerates and Aggregates as One Particle Each—CR2

**Figure 3:**
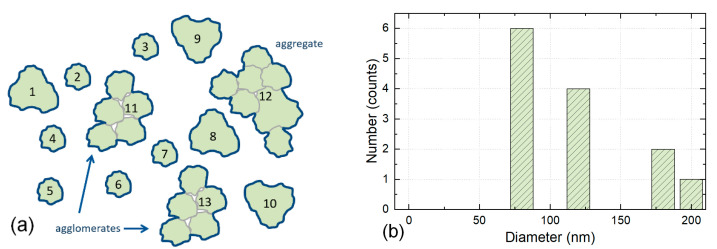
Illustration of Counting Rule 2. (**a**) The particles 1 to 10 are counted as individual particles—as in CR1. The agglomerates 11 and 13 and the aggregate 12 are counted each as one particle. This results in a total count of 13 particles. (**b**) The size of the agglomerates and the aggregate is 180 nm and 200 nm, respectively. Note the shift of the PSD to higher values in comparison to CR1. The height of the four columns in the histogram reflects the number of particles of the four sizes 80 nm (6 particles), 120 nm (4 particles), 180 nm (2 particles) and 200 nm (1 particle). The width of the columns has no quantitative relevance and is only illustrative.

In addition to the individual particles (see CR1), each aggregate and each agglomerate are counted as one particle, see Figure 3.

Analysis using EM methods cannot distinguish which agglomerates originate from the preparation of the specimen and which agglomerates were already part of the dispersion. According to this counting rule, both types of agglomerates are therefore counted, and each one is counted as one particle. Applying CR2 to EM measurements would lead to different outcomes compared to other characterisation methods, which measure the liquid suspension directly and thus do not need the step of preparing a specimen. This counting method is used in ISO 14887 [32].

### 3.3. Counting Individual and Touching Particles (within Agglomerates) as One Particle—CR3

Individual particles, touching particles, and integral components in agglomerates are each counted as a particle. Aggregates are counted as a particle.

**Figure 4:**
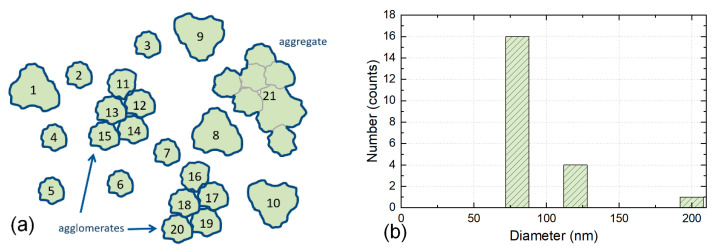
(**a**) Illustration of Counting Rule 3. The particles 1 to 10 are counted as individual particles. The particles which are part of the two agglomerates are counted as touching particles 11 to 15 and 16 to 20. The aggregate 21 is counted as one particle. This results in a total count of 21 particles. (**b**) Note the clear shift of the PSD towards smaller sizes. The height of the three columns in the histogram reflects the number of particles of the three sizes 80 nm (16 particles), 120 nm (4 particles), and 200 nm (1 particle). The width of the columns has no quantitative relevance and is only illustrative.

The preparation of the specimen on a substrate often leads to agglomeration on the surface of the substrate. Particles are clumped together, and they lay on the surface and can be identified; see the agglomerates sketched in Figure 4a. Since the integral components in agglomerates can be identified, according to CR3 touching particles are counted as individual particles. In most cases, there will be still some agglomerates in which the integral components cannot be identified. Those agglomerates and all aggregates are counted as one particle in CR3. This counting method is used in ISO 21363 [24] and ISO 19749 [33].

### 3.4. Counting of Particles within Agglomerates and Aggregates—CR4

**Figure 5:**
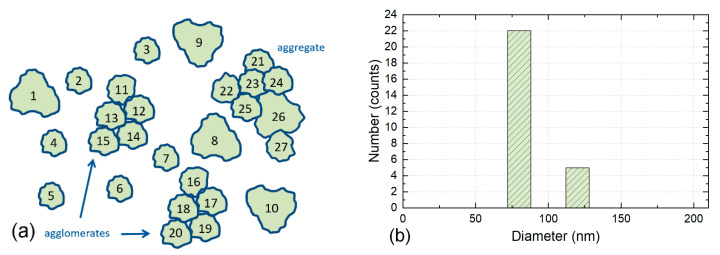
Illustration of Counting Rule 4. (**a**) The particles 1 to 10 are counted as individual particles. The integral components of the two agglomerates are counted as particles 11 to 15 and 16 to 20. In the aggregate the integral components are counted as particles 21 to 27. This results in a total count of 27 particles. (**b**) Note the shift of the PSD to lowest size values. The height of the two columns in the histogram reflects the number of particles of the two sizes 80 nm (22 particles) and 120 nm (5 particles). The width of the columns has no quantitative relevance and is only illustrative.

Individual particles and integral components in agglomerates and aggregates are each counted as one particle, see Figure 5.

Agglomerates and aggregates are examined for their integral components, and those components are counted. The integral components need to be either fully visible, or at least it should be possible to clearly determine the size of the component, represented, e.g., as minimum Feret diameter, equivalent circular diameter or maximum Feret diameter. There is currently no standard available to count particles within agglomerates and aggregates in accordance with this counting method.

This counting method is required to identify nanomaterials for regulatory purposes in the EU and to decide whether a material is a nanomaterial according to the EC’s recommended definition of nanomaterials. It is also required for risk assessment of nanomaterials to be applied in the food and feed chain [13] and to establish the presence of small particles, including nanoparticles, in regulated food and feed product applications [34].

The four counting rules proposed here cover a wide range of cases. Still, other counting rules may be defined, but the rules listed above will capture the majority of all counting cases encountered in practice. These theoretical approaches can therefore be regarded as a starting point for the counting of particles when analysing EM images to obtain results useful in contexts such as ISO and for legislation. Table 2 compares the application of these four different counting rules to the same hypothetical particles, which are counted in Figure 2, Figure 3, Figure 4 and Figure 5. As seen from the table, the number of counted particles, as well as the particle sizes, differ widely between the counting rules.

The above description demonstrates how different counting rules may significantly affect the final result of the particle size measurement. This knowledge is of paramount importance for the future development of automated counting of particles recorded on EM micrographs. Additionally, when regulatory obligations necessitate particle counting resulting in a regulatory classification (e.g., as a nanomaterial), the utmost care has to be taken to select the appropriate counting rule. As illustrated by the examples above, depending on the applied counting rule, the median value can be below and above 100 nm using the same images for analysis.

## 4. Counting of Particles in Practice

**Figure 6:**
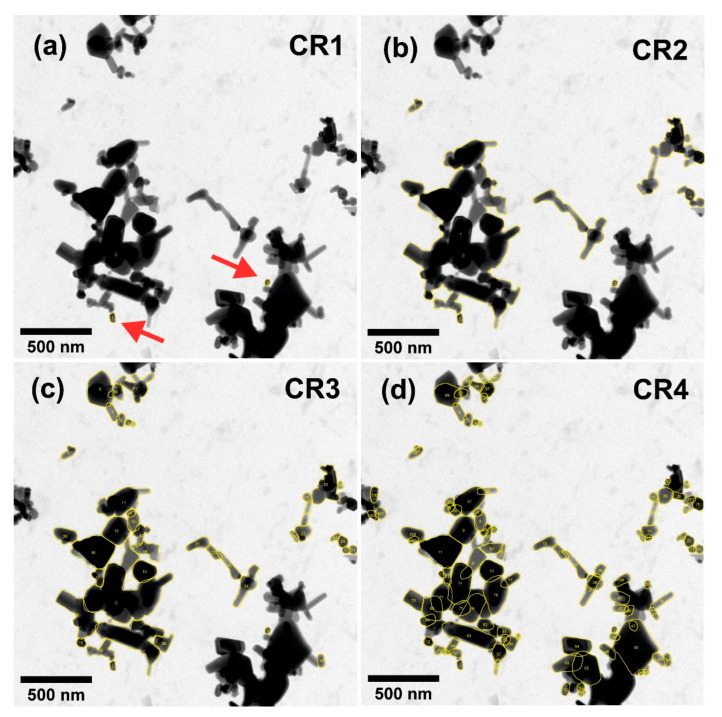
TEM image of overlapping zinc oxide particles with the particles marked according to the applied counting rule (**a**) CR1: 2 particles (pointed at by the red arrows), (**b**) CR2: 7 particles, (**c**) CR3: 41 particles, and (**d**) CR4: 104 particles. Counted particles have been given a yellow border. See Figures S1–S3 for the corresponding particle size distributions.

**Figure 7:**
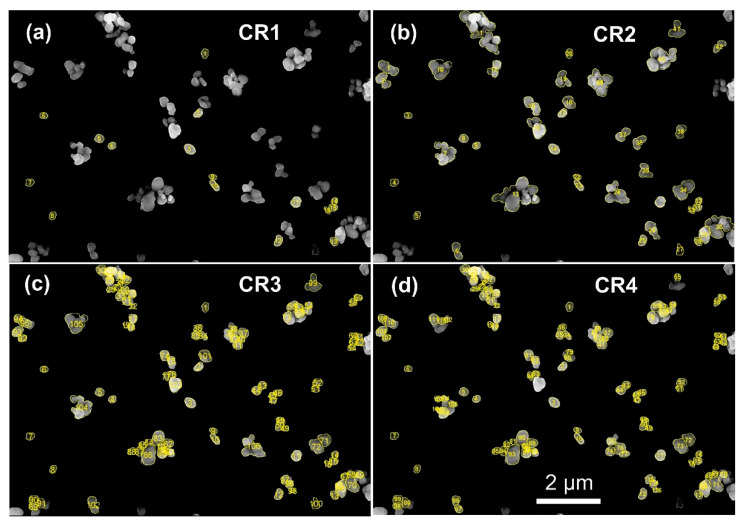
SEM image of titanium oxide particles prepared on a silicon substrate with the particles marked according to the applied counting rule (**a**) CR1: 16 particles, (**b**) CR2: 42 particles, (**c**) CR3: 106 particles, and (**d**) CR4: 116 particles. Counted particles have been given a yellow border. See Figures S4–S6 for the corresponding particle size distributions.

**Figure 8:**
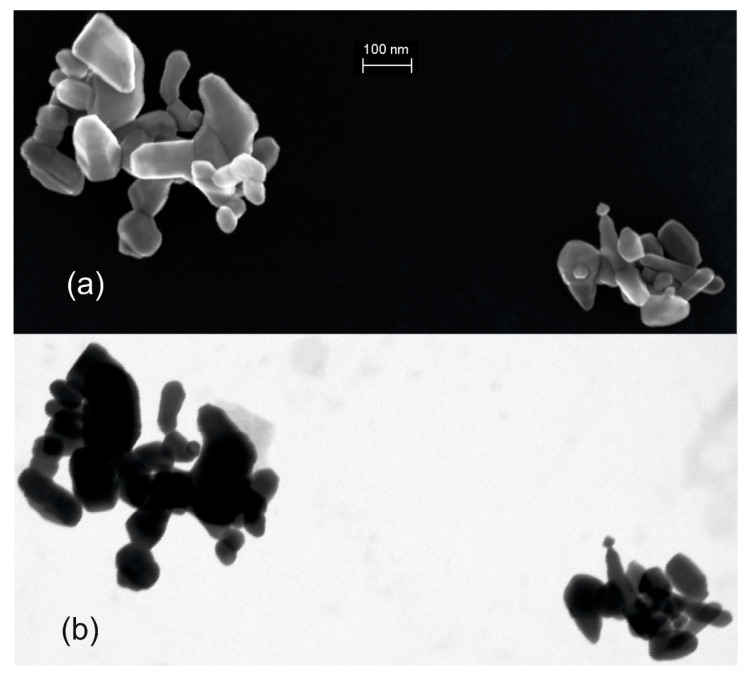
(**a**) SEM (SE InLens) micrograph and (**b**) STEM-in-SEM micrograph with the instrument in transmission mode of the same field-of-view as in (**a**) of two zinc oxide agglomerates/aggregates deposited on a carbon TEM grid.

In the previous chapter, we defined four different rules for the counting of particles. In practice, there are also cases where the rules cannot easily be applied or cannot be applied at all. Examples are shown in Figure 6 and Figure 7, where most particles are overlapping so that many particles are partly (or possibly completely) hidden. Some of the (partly) hidden particles can relatively clearly be still identified and measured; however, many of them cannot be identified unambiguously as individual particles. This is the main reason why the PSD of complex, agglomerated nanoparticles may not be representative for the whole particulate material in its initial state before an EM analysis. Often, smaller particles ‘stick’ to larger particles and Figure 8 illustrates that both in SEM (Scanning EM) (top view of the specimen surface) and TEM (transmission EM), and such fine particles, which are hidden beyond larger ones, cannot always be detected. Thus, a relevant fraction of the smaller-sized particles is not considered in the PSD, leading to an overestimation of the mean value of the PSD, and possibly to the false conclusion that the material is not a nanomaterial. Therefore, the ‘perfect’ dispersion of a polydisperse particulate material with only non-overlapping particles on a substrate is a prerequisite for a representative PSD (particularly with regard to the PSD ordinate, i.e., the particle number). This chapter will discuss some common cases and how to count the particles in accordance with the different counting rules.

Various size descriptors can be used depending on the specific analytical purpose of the measurement, e.g., for regulatory purposes in compliance with the EC nanomaterial definition that requires information on the smallest external dimension, which is best described by the minimum Feret diameter for particles with irregular shapes. More generally, when the size results measured via EM are compared with results obtained by other methods, which measure other measurands, e.g., the hydrodynamic diameter by DLS, the (area-)equivalent circular diameter (ECD) of the particles may be the more appropriate descriptor. For complex particle shapes, the determination of minimum Feret is simpler than the determination of the ECD, which assumes measuring the area of each particle accurately, resulting from the accurate identification of the particle boundaries. As described later with examples, in practice, there are almost always overlapping and touching particles present on the substrate, and an automated analysis based on unambiguous particle segmentation (i.e., separation from the image background) is challenging, i.e., prone to bias. Obtaining the true segmentation threshold for the identification of the entire boundary/contour of each particle needs very high expert knowledge. Hence, especially for complex particle shapes and a high degree of agglomeration/aggregation, the manual image analysis is still the current state, and in most cases the minimum or maximum Feret is measured as it is one of the simple but relevant sizes. When performing an analysis of a particle size distribution, one must be aware that the result can depend on the size descriptor used, and the more the particles deviate from a compact and regular shape and a narrow monodisperse size distribution, the stronger is this dependence. This becomes evident from the examples described in the following (cf. the particle size distributions in the Supplementary Information).

### 4.1. Sample Preparation for Electron Microscopy

All samples were prepared and analysed at BAM in accordance with the following procedures for dispersion, sonication and deposition on the substrate:

NM-110 ZnO (JRC). The material is distributed in powder form. A total of 0.16 mg of the powder was dispersed in 1 mL of ethanol. The sample was treated in a vial sonicator (Hielscher UP200St vial tweeter) for 15 min at 75% amplitude with a cycle time of 50% (1.2 W/mL). A total of 7.7 µL on the TEM grid. A total of 7.7 µL on the silicon wafer and spin coating with 2400 rpm for 60 s.

IRMM 388 coated TiO_2_ (JRC). The material is distributed in powder form. 0.15 mg of the powder was dispersed in 1 mL of ethanol. The sample was treated in a vial sonicator (Hielscher UP200St vial tweeter) for 15 min at 75% amplitude with a cycle time of 50% (1.2 W/mL). A total of 7.7 µL on the TEM grid. A total of 7.7 µL on the silicon wafer and spin coating with 2400 rpm for 60 s.

The specimens were prepared on a TEM grid (copper mesh with carbon film) for TEM. Specimens for SEM were prepared on a silicon wafer. Preparation has been performed by spin-coating or by drop deposition of the material dispersion. The wafers were not coated with a conductive material after sample deposition. Image evaluation was performed without any image preprocessing or thresholding by manual identification of the boundary of each particle and determination of the minimum Feret diameter, maximum Feret diameter and equivalent circular diameter of the particle. Mean and median diameters were measured under the assumption of a lognormal distribution. Further details about the image acquisition conditions can be found in the Supplementary Information.

### 4.2. Typical TEM Image with Different Counting Possibilities

Figure 6 shows a TEM micrograph of zinc oxide particles. Most particles overlap or touch each other, i.e., agglomerates or possibly aggregates are available, and only two individual particles can be identified. The application of CR1 to the micrograph leads to the identification of two individual particles, both being smaller than 100 nm, as the agglomerates and aggregates are excluded from the counting.

CR2 counts seven particles (with five of them being agglomerates or aggregates, which have a min Feret above 100 nm) and a mean particle size above 100 nm. Application of CR3 results in a number of 41 particles—mostly identified in the agglomerates—and CR4 leads to the identification of 104 particles, which include particles identified in aggregates.

When analysing the TEM micrograph in Figure 6, the identification of each contour of the particles was performed manually by drawing the contour with the mouse. Drawing the contour influences the results for the different CRs, depending on the operator. For CR1, we identified and counted two individual particles. Other operators might identify a different number of particles, potentially leading to a large deviation. For CR2 and CR3, roughly 20% of the deviation in particle number can be estimated. For CR4, the deviation can be estimated to be roughly 10–20%. For other materials, the deviations will vary.

For the particles of the materials selected and analysed in this paper, an asymmetry of the as-measured particle size distributions for different size descriptors has mostly been observed, with a longer tail on the right side (larger sizes), see SI. For this reason, a lognormal distribution function has been considered. However, other types of particles may result in other types of particle size distribution functions which match better the as-measured size descriptor distribution. The exact type of size distribution to be considered is not within the scope of this paper. For more information, see, e.g., the case studies in ISO 21363 [24].

Depending on the complexity of the shape of the particles identified, the mean value of the specific, measured size descriptor (min Feret, max Feret or ECD) of the particle size distribution can vary. The more “fragmented” the CR measures (increasing from CR2 to CR4), the lower the mean value of the particle size distribution will be, so CR2 and CR3 lead to mean sizes of 255 nm and 113 nm for the min Feret. In contrast, CR4 results in a mean value of the min Feret diameter below 100 nm (73 nm); see Table 3 and Figure S1 in Supplementary Information. With this example of mean or median values below and above 100 nm, it becomes evident that the applied counting rule significantly influences the classification of a material as a nanomaterial or not a nanomaterial. Figures S2 and S3 in the Supplementary Information illustrate the corresponding particle size distributions when ECD and max Feret are the size descriptors measured. Table 3 compares the results from applying the four counting rules to the same image (see the Supplementary Information), Figure 6, for several size descriptors. As can be seen from Table 3, both the counting rules and the descriptor reported can significantly influence the outcomes of the size measurement of the material. For the reporting of size, both pieces of information should be associated to allow a full understanding of the results.

It should be noted that, mostly, the unique identification of a certain type of particle (individual, agglomerate and aggregate) is impossible and that each operator identifies the particles in a subjective way; hence, the number of the identified particles may vary. However, of decisive importance for the traceability and reproducibility of the final result are (i) the specification of the counting rule applied and (ii) the exact documentation of the image analysis. The latter is fulfilled by marking/labelling the counted particles according to the applied rule in the archived images together with the values of the size descriptors measured on the counted particles.

### 4.3. Small Agglomerates with Identifiable Integral Compounds

Figure 7 shows the result of imaging titanium dioxide particles by SEM after preparation of the particles from liquid suspension on a silicon substrate. First, only about 16 individual particles are clearly identified on the substrate, and these would be counted according to CR1. Most particles are agglomerated in groups of 2–3 and up to about 10 or even more particles. When applying CR2, the number of counted particles will increase significantly (including all the agglomerates), and even more so when applying CR3 (including all identifiable integral components within the agglomerates). As no aggregates seem to be present, the outcome of applying CR4 will be similar to CR3. In all cases, the mean or median of the selected size descriptor will be at values above 100 nm. The Supplementary Information, Figures S4–S6, illustrate the corresponding particle size distributions when using min Feret, ECD and max Feret.

### 4.4. The Influence of EM Instrumentation and of the Specific Measurement Method

One sees in an electron micrograph what the instrument can measure and what the operator adjusts to record the image. In other words, the quality of the information visible in a micrograph is strongly dependent on the performance of an instrument and the settings selected by the operator. A high-end SEM equipped with high-resolution cathode and detectors is able to visualise fine nanoparticles of a few nm; other instruments (e.g., table-top SEMs) can barely detect objects below 100 nm. Furthermore, some electron detectors are very sensitive to surface morphology, and there are detection ways that are more suitable for mass-thickness analysis. Such an example is given in Figure 8, where different operating modes of the same SEM instrument enable different information levels of the same field of view of the specimen. Thus, Figure 8a shows a micrograph of a specimen recorded with the surface-sensitive SE InLens detector, which enables the identification of more structural information of the two agglomerates or aggregates than in the transmission mode in Figure 8b. Whilst for the STEM-in-SEM [35] micrograph in Figure 8b, CR1 results in zero particles, and CR2 to CR4 result in basically two particles, the surface-sensitive SEM micrograph in Figure 8a also leads to zero particles by CR1, and definitely more particles by CR2 to CR4, due to the ability to clearly distinguish integral components in the two agglomerates/aggregates.

More information about the different working modes of an electron microscope linked to their corresponding performance regarding high-resolution imaging capabilities, advantages and disadvantages can be found in dedicated systematic reports in the literature. Examples are [35,36] for STEM-in-SEM (or TSEM), highlighting the superior material contrast and the possibility of more accurate particle boundary extraction, [35,37,38] for SEM with evidence of particle size overestimation due to high surface and charging sensitivity (particularly the SE InLens detector), or [1,35,39] for TEM on agglomerated and aggregated nanoparticles, where relatively small size diffraction effects might obscure the real particle boundaries or where HAADF-STEM is preferred over the bright field imaging. For the appropriate use of the right imaging mode in all these cases, deep expertise is necessary to evaluate the true particle boundaries accurately and thus the true size of the particles.

### 4.5. Beyond the Limits of EM

**Figure 9:**
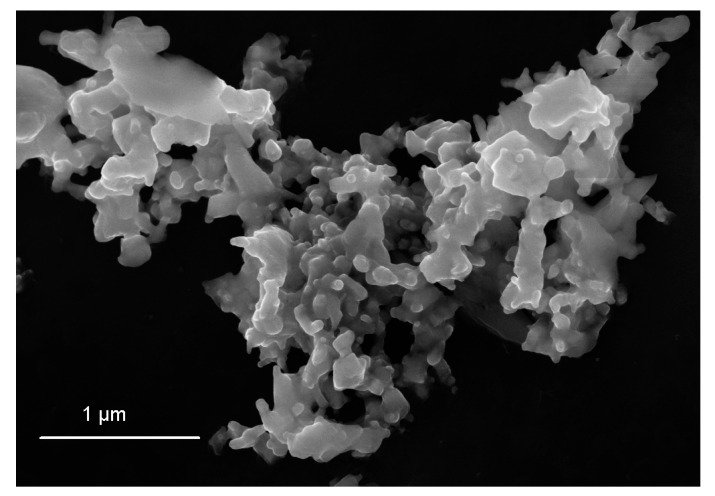
SEM micrograph of zinc oxide showing a large and complex agglomerate/aggregate.

As already highlighted above, features which are smaller than the highest achievable resolution of an EM cannot be detected. Figure 9 presents a micrograph in which it is not clear whether integral components can be identified or not, i.e., no clear separation of particles within agglomerates/aggregates is visible. Based only on this image, the result of the application of CR1 is zero individual particles, and of all the other three counting rules, CR2 to CR4 is one particle. One option to attain more information on the structures partly visible within the large agglomerate/aggregate is either to change the analysis method (other imaging modes, possibly 3D, or a combination of non-imaging methods) or to try to improve the sample preparation procedure used. It is possible that residues of the solvent in the initial suspension with the particulate material to be analysed are still present around/between the potential integral components partly indicated within the agglomerate/aggregate in Figure 9. A longer drying time might help to eliminate any remaining moisture.

### 4.6. Proposed Procedure for a Reliable Counting of Small Particles

Based on the information above, we propose the following steps for a reliable counting of small particles, including nanoparticles, with EM. Before starting on the sequence outlined below, it might be useful to obtain a descriptive micrograph of the material.

(a) Identify the goal of the characterisation (quality control, registration in accordance with specific regulatory requirements, etc.).
(b) Identify and name the relevant counting rule (e.g., CR1, CR2, CR3, CR4) in accordance with possible needs stated by the scientific community, standardisation (e.g., ISO) or legislation.
(c) Identify and apply an appropriate dispersion protocol for the material and an appropriate protocol for the sample preparation. Be aware that the chosen protocol will influence the characteristics of the dispersion and thereby also lead to different results. An optimal and reproducible dispersion and sample preparation are crucial for reliable results.
(d) Identify, measure and count all individual particles which are not bound to agglomerates or aggregates and are not overlapping.
(e) Identify, measure and count all particles that are touching or overlapping but the borders can be identified with high confidence and accuracy (Examples see Section 4.2 and Section 4.3).
(f) Overlapping particles where the borders cannot be identified with high confidence should be regarded as agglomerates and identified, measured and counted in accordance with the relevant counting rule.
(g) Fused particles with inaccurate borders should be regarded as aggregates and identified, measured and counted as such in accordance with the relevant counting rule.
(h) Where it is not possible to identify borders between the particles, this particle should be counted in accordance with the appropriate counting rule or excluded from the analysis.

Throughout this procedure, the operator(s) must keep track of and record all metadata and actions, including detailed and individual marking of all identified and counted particles in the evaluated micrographs. This is the only way for the transparent and reproducible evaluation of EM data regarding the distribution of particle size.

## 5. Conclusions

Four ways, ‘counting rules’, for the reliable counting of particles recorded on EM-images have been identified from the literature. The counting rules are explained and, importantly, possible outcomes of applying them to the same EM micrographs are compared in this paper. The examples represent a range of cases from easy ones with a low degree of agglomeration and clearly identifiable particles up to particles of complex shape and a high degree of agglomeration and aggregation, and a final example of a fully aggregated, “unresolvable” material. In simple cases, e.g., when the sample/specimen consists of only individual particles, all CRs will deliver the same number. However, in most real-life cases, the number of particles counted and their size differ significantly between the counting rules. Hence, using the outcome of these rules to determine whether a material is a nanomaterial or not may lead to different conclusions depending on the exact rule applied. Especially depending on how agglomerates and aggregates are counted, the results might differ significantly. This is important for the classification of a particulate material as nanomaterial or not, and it highlights the importance of applying a defined counting rule with the evaluation of the measurement as well as stating which rule was applied. This is illustrated in Table 3, where an overview of the outcomes of applying the four counting rules to the same TEM micrograph (shown in Figure 6) is presented. Table 3 clearly shows that, depending on the counting rule applied and the descriptor reported, the number of particles counted varies from 2 to 104, and the diameter from 43 nm to 413 nm.

The examples given are deliberately selected so that slight variations in the counting rule can have significant variations of the main size descriptors with the consequence that depending on the (i) applied counting rule and (ii) measured size descriptor, the same particulate material from the same EM images can be categorised as a material with a size above or below a selected threshold (e.g., 100 nm).

The four different counting rules for particles are all applied in science, and beyond this, they are needed for regulatory identification of nanomaterials for which the legislation may define which counting rule to apply. It is thus of utmost importance, also in a regulatory context, to be fully aware of which particles are counted and how they are counted according to each of these rules.

Furthermore, we have illustrated the practical evaluation of EM-images, noting that even if, in theory, the counting is clearly defined, identifying and separating particles on an image is a subjective task which increases the uncertainty of the results. Depending on the material under examination, it may even be impossible to obtain meaningful EM micrographs.

Throughout any analysis of particles, including the evaluation of electron micrographs, it is essential to keep track of and record all metadata and actions, including detailed and individual marking of all identified and counted particles in the evaluated micrographs. This is the only way for the transparent and reproducible evaluation of EM data regarding the distribution of particle size.

While EM is the gold standard, it is not always possible to use EM for the characterisation of a material (compare Figure 9). For such particles, it should be considered to determine the size distribution by an appropriate combination of other methods than EM or a combination of these. It should also be noted that EM is not a suitable technique for some materials under certain analysis conditions, as, e.g., the electron beam might damage some electron beam- or vacuum-sensitive materials.

In this paper, we have identified and described four different counting possibilities for particles recorded in EM images. These counting rules support the application of OECD test guidelines, in which the actual ways of counting particles are often not described in detail, as well as the application of ISO standards. The four rules are also helpful for understanding regulatory provisions which require the reporting of particle size distributions. It should also be noted that this detailed knowledge of the counting rules is of paramount importance for future applications in automated counting algorithms. Currently, there are no established software packages or algorithms available that are validated for the counting of (nano)particles, including agglomerates and aggregates.

## Figures and Tables

**Table 1:** Comparison of the essential elements of the ISO and EC definitions for the term “constituent particle”.

**Constituent Particle** In order to understand the exact meaning of “constituent” in a specific context, the point of reference is crucial, i.e., the “unit” or “whole” to which it refers.	
**ISO Definition**	**EC Definition**
The point of reference are larger particles or “units”, such as agglomerates and aggregates.	The point of reference is the initial material, or “whole” material.
According to ISO 80004-2, a constituent particle is an “identifiable, integral component of a larger particle” [26].	The EC definition describes the term nanomaterial: ” ‘nanomaterial’ … should be based solely on the size of the constituent particles of a material…” and continues further explaining what these constituent particles are: “ ’Nanomaterial’ means a … material containing particles, in an unbound state or as an aggregate or as an agglomerate…” [16].
Accordingly, constituent particles are always part of a larger ensemble and can be aggregates themselves, which could form even larger agglomerates.	The term “constituent particle” refers to the entire material, so individual particles as well as the constituents of agglomerates and aggregates are considered constituent particles of a material.

**Table 2:** Comparison of the outcomes of applying different counting rules to the same set of hypothetical particles and in the same images (see Figure 2, Figure 3, Figure 4 and Figure 5).

CR	Total Number of Particles	No. of Particles of a Specific Size			
		80 nm	120 nm	180 nm	200 nm
**CR1**	10	6	4	0	0
**CR2**	13	6	4	2	1
**CR3**	21	16	4	0	1
**CR4**	27	22	5	0	0

**Table 3:** Comparison of descriptor values and results when applying the Counting Rules to the TEM micrograph of zinc oxide shown in Figure 6. The numbers are obtained assuming a lognormal distribution. See Figures S1–S3 for details of the corresponding particle size distributions.

d_mean_; d_median_; SD * (nm)					
	Counting Rule	CR1	CR2	CR3	CR4
Descriptor					
Minimum Feret diameter		43; 43; ±1	255; 98; ±109	113; 85; ±13	73; 58; ±4
ECD **		52; 52; ±6	242; 126; ±105	127; 106; ±13	94; 78; ±5
Maximum Feret diameter		64; 64; ±11	413; 215; ±166	182; 138; ±21	124; 99; ±7
Number of particles		2	7	41	104

* Standard deviation; ** Equivalent circular diameter.

## Data Availability

Data can be provided by the authors on request.

## Supplementary Materials

The following supporting information can be downloaded at: https://www.mdpi.com/article/10.3390/nano12132238/s1, (a) Overview page of counting rules. (b) Figures S1–S3: Figures illustrate the corresponding particle size distributions for zinc oxide (Figure 6) when using min Feret, ECD and max Feret. (c) Figures S4–S6: Figures illustrate the corresponding particle size distributions for titanium dioxide (Figure 7) when using min Feret, ECD and max Feret. (d) Report details.
