# Supplementary material for: Counting Small Particles in Electron Microscopy Images—Proposal for Rules and Their Application in Practice
Source: Nanomaterials (Basel). 2022 Jun 29;12(13):2238. doi: 10.3390/nano12132238 (PMC9268650; doi:10.3390/nano12132238)
Supplement: Supplementary file 1 [file nanomaterials-12-02238-s001.zip › nanomaterials-1774246-supplementary.pdf]

Supplementary Materials

Counting Small Particles in Electron Microscopy Images—Proposal for Rules and Their Application in Practice

Harald Bresch [1,*], Vasile-Dan Hodoroaba [1,*], Alexandra Schmidt [1], Kirsten Rasmussen [2] and Hubert Rauscher [2]

[1] Federal Institute for Materials Research and Testing (BAM), D-12200 Berlin, Germany; schmidtalexandra63@gmail.com (A.S.)
[2] Joint Research Centre (JRC), European Commission, 21027 Ispra, Italy; kirsten.rasmussen@ec.europa.eu (K.R.); hubert.rauscher@ec.europa.eu (H.R.)
* Correspondence: harald.bresch@bam.de (H.B.); dan.hodoroaba@bam.de (V.-D.H.)

Overview of Particle Counting Rules

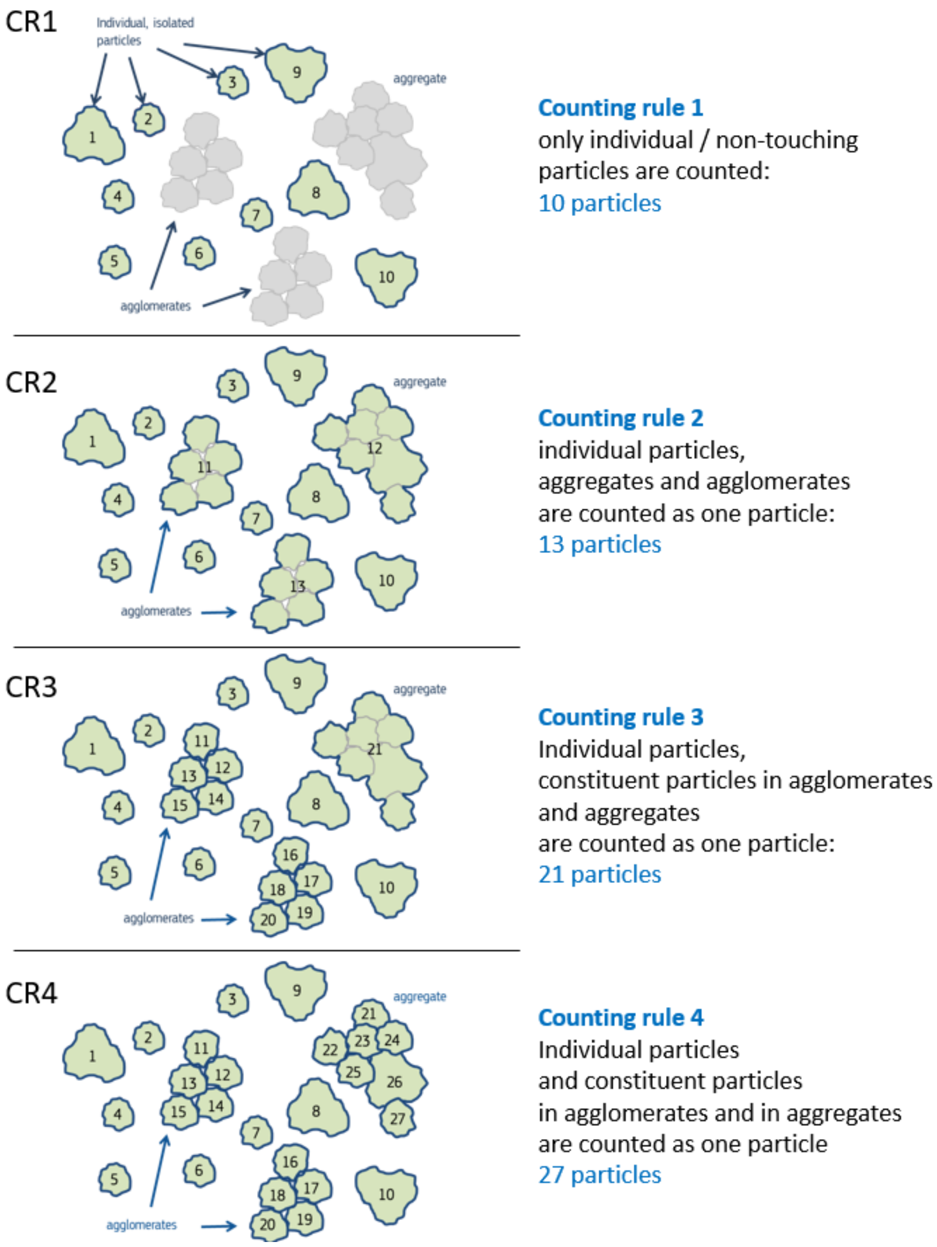

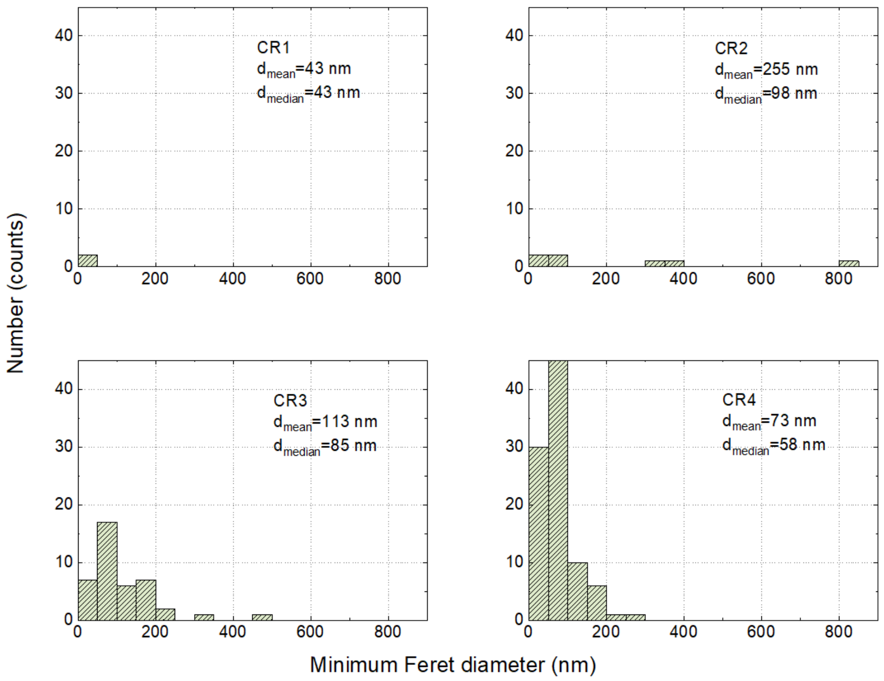

Figure S1. Minimum Feret Diameter of zinc oxide based on the application of the CR1 to CR4 to the TEM image in Fig. 6. As seen also from these graphs the number of particles counted varies significantly with counting rule applied as does the mean and median size of these particles.

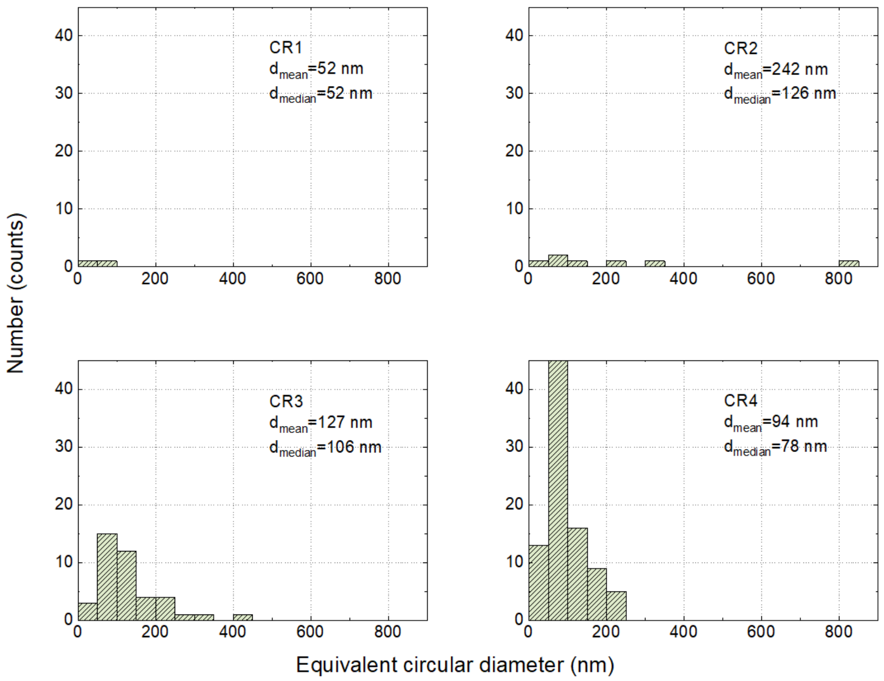

Figure S2. Equivalent circular diameter of zinc oxide as resulted after the application of the CR1 to CR4 to the TEM image in Fig. 6. As seen also from these graphs the number of particles counted varies significantly with counting rule applied as does the mean and median size of these particles.

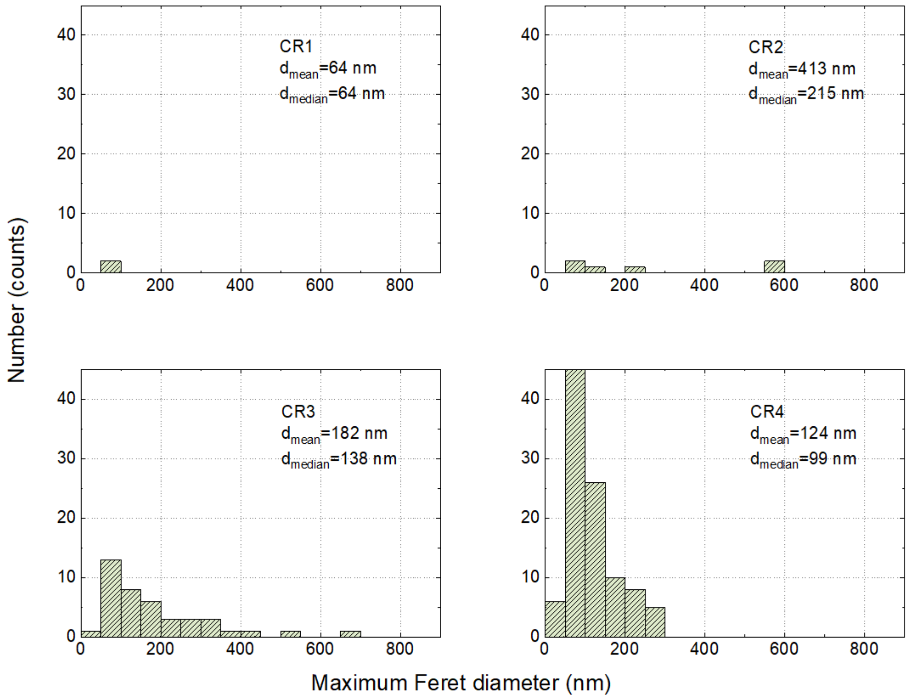

Figure S3. Maximum Feret Diameter of zinc oxide based on the application of the CR1 to CR4 to the TEM image in Fig. 6. As seen also from these graphs the number of particles counted varies significantly with counting rule applied as does the mean and median size of these particles.

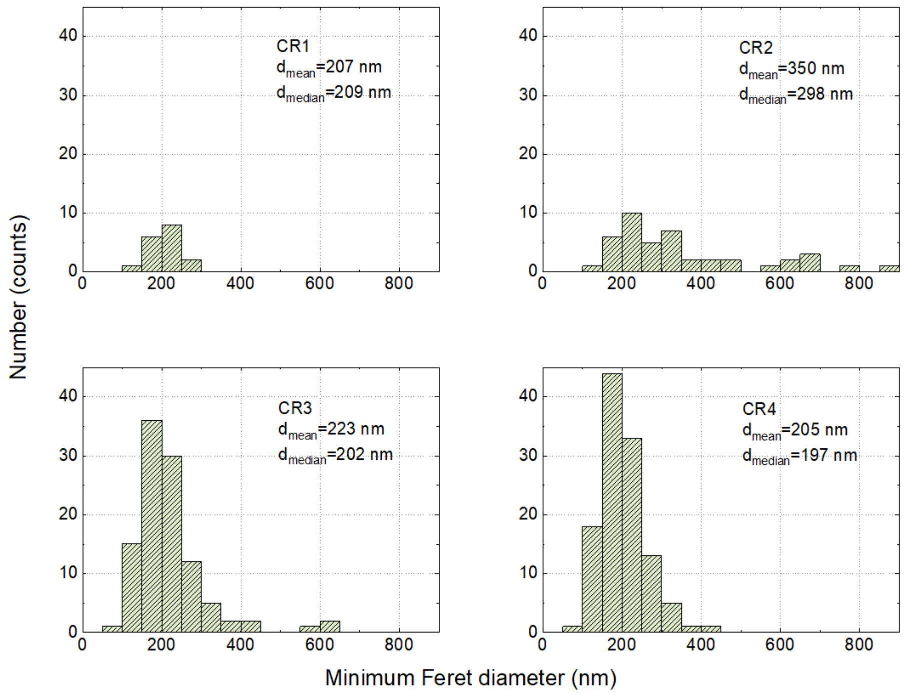

Figure S4. Minimum Feret Diameter of titanium oxide obtained after the application of the CR1 to CR4 to the TEM image in Fig. 7.

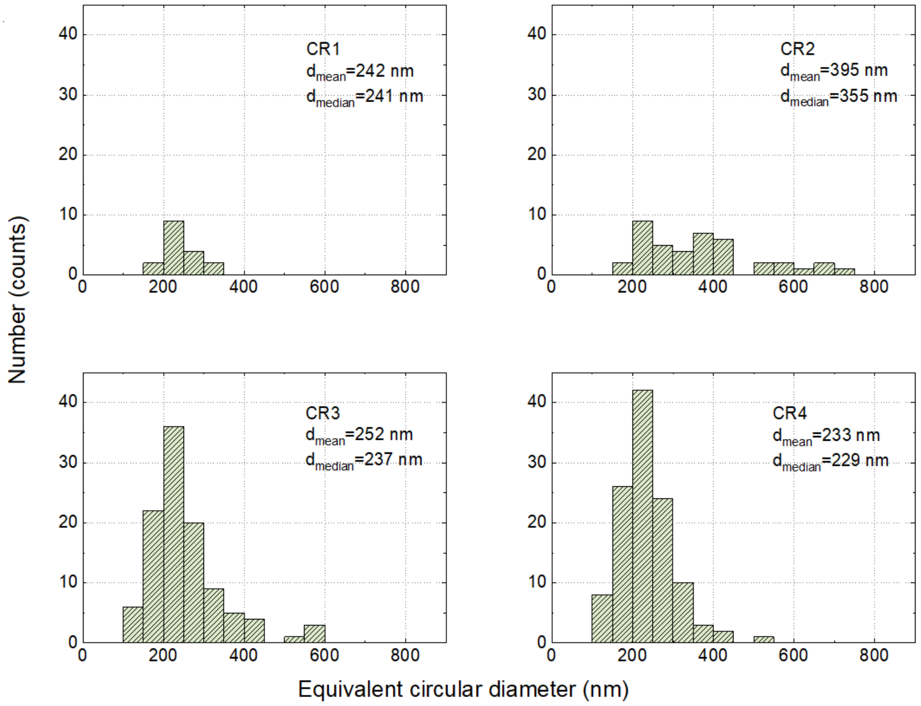

Figure S5. Equivalent spherical diameter of titanium oxide obtained after the application of the CR1 to CR4 to the TEM image in Fig. 7.

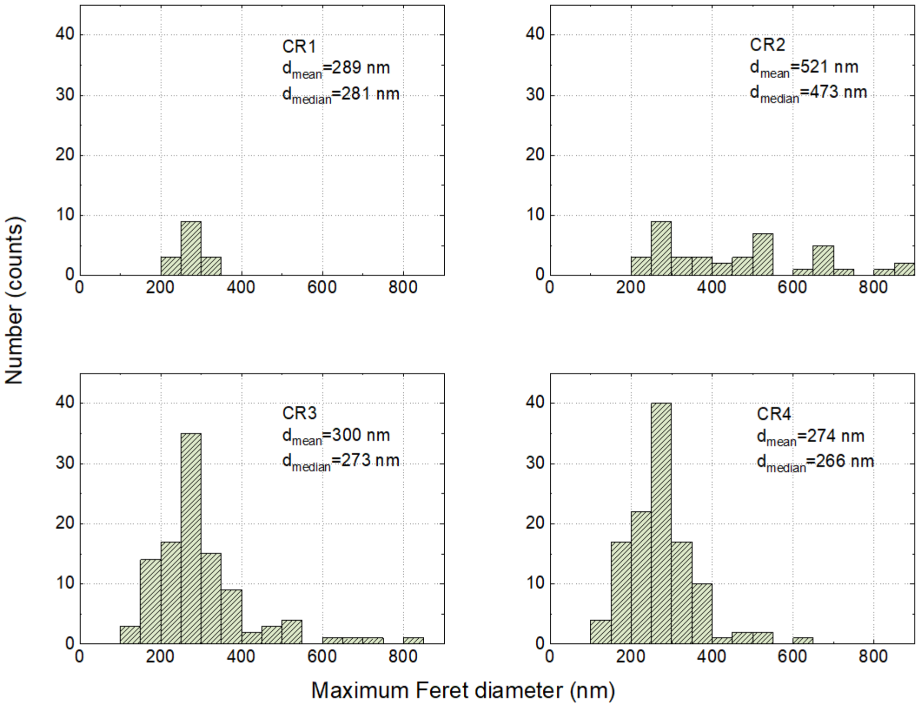

Figure S6. Maximum Feret Diameter of titanium oxide obtained after the application of the CR1 to CR4 to the TEM image in Fig. 7.

Table S1. compares descriptor values and results when applying the Counting Rules to the TEM micrograph (titanium dioxide) shown in Figure 7. The numbers are obtained assuming a lognormal distribution. See Figures S4–S6 for details of the corresponding particle size distributions.

d_{mean}; d_{median}; SD (nm)				
**Descriptor \ Counting rule**	**CR1**	**CR2**	**CR3**	**CR4**
**Minimum Feret diameter**	207; 209; ± 11	350; 298; ± 29	233; 202; ± 9	205; 197; ± 5
**Equivalent circular diameter**	242; 241; ± 10	395; 355; ± 30	252; 237; ± 9	233; 229; ± 6
**Maximum Feret diameter**	289; 281; ± 12	521; 473; ± 46	300; 273; ± 12	274; 266; ± 8

Information on image acquisition and used software

Image acquistion:
Titanium dioxide (Figure 7)
Instrument: ZEISS Gemini Supra 40
Electron beam voltage: 10 kV
Pixel size: 4.9 nm
Type of detector: SE InLens

Zinc oxide
Figure 6:
Instrument: JEOL JEM-2200FS
Electron beam voltage: 200 kV
Pixel size: 0.853 nm
Type of detector: Gatan CCD camera

Figure 8:
Instrument: ZEISS Gemini Supra 40
Electron beam voltage: 20 kV
Pixel size: 1.5 nm
Detector type: i) SE InLens and ii) conventional SE detector applied to the transmitted electrons via a dedicated sample holder (STEM-in-SEM)

Figure 9:
Instrument: ZEISS Gemini Supra 40
Electron beam voltage: 10 kV
Pixel size: 4.9 nm
Type of detector: SE InLens

Software:
ImageJ 2.1.0/1.53c
OriginPro2022
